# The London Hospital Nurses

**Published:** 1919-01-11

**Authors:** 


### THE EDITOR'S LETTER-BOX.

### The London Hospital Nurses.

*To the Editor of* THE HOSPITAL.

SIR,—After great difficulties I have been able to communicate with Miss Helen McKinley, and I append a letter I have received from her to which I am sure you will give the same publicity you gave to her original statement.—Yours, etc.,

KNUTSFORD.

61 Lowndes Square, S.W. 1, January 1, 1919.

